# Examining User’s Initial Trust Building in Mobile Online Health Community Adopting

**DOI:** 10.3390/ijerph17113945

**Published:** 2020-06-02

**Authors:** Yuanyuan Cao, Jiantong Zhang, Liang Ma, Xinghong Qin, Junjun Li

**Affiliations:** 1 School of Management, Hangzhou Dianzi University, Hangzhou 310038, China; cyy2013@hdu.edu.cn (Y.C.); admire185@126.com (J.L.); 2 School of Economics & Management, Tongji University, Shanghai 200092, China; 3 Management Science and Engineering, Shandong University of Finance and Economics, Jinan 250014, China; maliang1010@126.com; 4 School of Management Science and Engineering, Chongqing Technology and Business University, Chongqing 400067, China; qinxinghong4515@sina.com

**Keywords:** mobile online health community, initial trust, elaboration likelihood model, trust transfer, performance-based cue, transfer-based cue, adopting intention

## Abstract

Due to the high perceived risk, it is critical to foster users’ initial trust in the promotion of mobile online health community (MOHC) adoption. The present study focused on the role of two different trust elements and examined the initial trust building process based on elaboration likelihood model and trust transfer theory. The results indicated that initial trust in MOHC context was composed of two interrelated components: health service provider (doctor) and underlying technology (MOHC platform). Especially, the initial trust in MOHC platform exerted greater effects on adopting intention. Both performance-based cue (doctors’ information quality and interaction quality) and transfer-based cue (trust in the offline doctors’ health service) positively shaped the initial trust in doctor. Meanwhile, only the performance-based cue (MOHC platform’s information quality and service quality) has significant positive association with initial trust in MOHC platform. However, interpersonal recommend is insignificantly related to the initial trust in doctor. Trust in the mobile internet service is insignificantly related to the initial trust in MOHC platform.

## 1. Introduction

Mobile online health community (MOHC) reshapes the traditional health information acquisition mode. MOHC expands offline medical services to online and provides professional medical health consultation, which guide patients to choose a doctor more accurately and reduce blindness [1]. It helps users to access medical consultation service anytime and anywhere without the limitation of time and space. Despite MOHC providing great convenience to users, its penetration and adoption is relatively low [2,3,4]. A case study on “Hao Daifu Zaixian”, a well-known and representative MOHC platform in China, indicated that less than 1% visitors have used the mobile medical health service [1]. The lower adopting rates hinder the diffusion of the MOHC, which is a disadvantage in solving the common people’s medical treatment difficulty and optimizing medical resources distribution. Acquiring users and facilitating their adoption is the first step for the success of emerging MOHC. Thus, it is important to explore the factors that affect MOHC adoption and take effective measures to facilitate users’ acceptance and usage.

Due to the important role and advantages of MOHC in alleviating social medical pressure, related research on MOHC user behavior has received attention from scholars. For example, some researchers examined the knowledge sharing behavior, switching behavior and continuous participation behavior under the context of MOHC [5,6,7,8]. Although the existing research helps us to understand the user behavior in the MOHC environment, there are still some research gaps. (1) The existing MOHC user behavior research seldom focuses on adopting behaviors. (2) Although prior studies of mobile health product adopting behavior have already been based on UTAUT, TAM, VAB and TPB to explore technological factors, individual characteristics, value factors, environmental factors and psychological factors in mHealth user adoption behavior [2,4,9,10,11,12,13,14], the effect of initial trust in the adopting behavior has been seldom explored (detailed influence factors are shown in Table 1). The existing adopting research result on general mobile health products cannot comprehensively explain the adopting behavior in the special research context of MOHC. As a subcategory of mobile health products, different from ordinary mobile health products, MOHC is a profession medical consultation platform. The information and service provided by MOHC is directly related to the life and health of the user, which involves great risk. Building initial trust is crucial for alleviating the perceived risk and decreasing the perceived uncertainty in the earliest interaction stage between users and information system (IS) [15]. Initial trust determines users’ first time using [16,17] and further influences their continuance intention [18]. Therefore, it is important to explore the MOHC adopting behavior from the initial trust perspective.

Elaboration likelihood model (ELM) provides an appropriate theory framework for the present study, which has been widely used in trust research in various contexts. Meanwhile, based on the character of MOHC, we integrate the ELM with trust transfer theory to explore the initial trust building process. The novelty of our study is as follows: (1) it is different to prior trust studies, which mainly focused on overall trust and ignored detailed trust elements. We examine two dependent elements’ initial trust building process and the relationship between these two elements. As a professional patient-to-doctor medical health consultation platform, the operation of MOHC depends on multiple elements, such as health service provider (doctor) and underlying technology (MOHC platform). Each of these specific elements could invoke distinct trust beliefs and influence the user’s initial trust level. (2) Contrasting with prior studies, which had seldom simultaneously considered performance-based [19] and transfer-based [20,21,22] trust building paths, the present study integrates these two trust building paths in ELM. In the central route, we mainly examine the performance-based cue, which requires more cognitive effort [19], such as information-related arguments. In the peripheral route, we mainly examine the simple heuristic cues, which requires a less cognitive effort [19], such as trust transfer-based cue. Thus, in general, the objective of this study is to explore the following two questions:

(1) In the provision of MOHC service, how are the different initial trust elements interrelated and further influence users’ adoption?
(2) For different initial trust elements in the MOHC, how are they formed based on the performance-based and transfer-based, respectively?

In order to explore these research questions, an empirical study was conducted in China. In recent years, under the promotion of government policies, various MOHC platforms sprang up in China, such as “Hao Daifu Zaixian” and “Chunyu Doctor”. In China, MOHC is just in the initial development stage and has not been widely adopted, which just fits the research background of the present study. Thus, the research is carried out based on the case of China. Meanwhile, MOHC includes two types: patient-to-patient community and patient-to-doctor community [23]. We mainly focus on patient-to-doctor community, which provides a bridge for the doctors and patients online. The contribution of this study is as follows: Firstly, the present study extends the MOHC user behavior literature and mHealth adopting study by examining the MOHC user’ adopting intention from the perspective of initial trust. Secondly, the findings of the present study will enrich the MOHC user behavior research by identifying the different initial trust elements role in users’ MOHC adopting intention. Thirdly, this study simultaneously examines the transfer-based and performance-based initial trust building paths, which will contribute to the research of trust in the mobile health field. The finding of the present study also provides managerial implications for MOHC service providers and the medical management department of government on how to promote the adopting of MOHC. 

The structure of the rest of the paper is as follows. In Section 2, the related literature about the online health community user behavior, initial trust, trust transfer and ELM theory is reviewed. Then, in Section 3, theoretical model and relative hypotheses are developed. In Section 4, the method of this research is described. In Section 5, the data analysis process and results are presented. In Section 6, the key finding from the result, theoretical and managerial insights are presented. In Section 7, the limitations and future research directions are finally presented. 

## 2. Literature Review

### 2.1. Online Health Community User Behavior

As an emerging online health service, the scholars have already payed attention to online health community and examined the user behavior in it. Zhang et al. (2017) studied the effect of extrinsic and intrinsic motivation on professionals and normal users’ sharing behavior [8]. Li et al. (2019) revealed the effect of different types of information on patient’s switching behavior from a doctor’s online medical service to offline [7]. Based on the stimulus–organism–response theory, Zhou (2019) examined the relationship among privacy concern, trust and motivations underlying the users’ knowledge sharing intention in online health community context [5]. Liu (2020) examined the effect of perceived community support on user interactions and value co-creation, which further affects continuous participation [6].

Mobile online health community is a sub-category of mobile health. Although mobile health adoption behavior has been widely investigated, few studies have segmented the mobile health products and focused on MOHC adoption. Table 1 lists the relevant influence factors regarding the mobile health adopting, which had been investigated in the prior studies of recent years. The influencing factors can be divided into the following categories: (1) technological factors such as perceived ease of use and perceived usefulness; (2) individual characteristics such as technology anxiety, resistance to change, health stress and gender; (3) value factors such as price value, context value and content value; (4) environmental factors such as social influence and facilitating conditions. (5) psychological factors such as attitude, performance expectancy and effort expectancy [2,4,9,10,11,12,13,14]. Different from general mHealth product, the adoption of MOHC is not only limited to the above factors, which have been examined in the prior studies. MOHC is a complex social-technological system, and establishing and cultivating the initial trust of users is particularly important for the adopting and long-term development of MOHC.

As evidenced by these studies, the previous researches have studied the knowledge sharing behavior, switch behavior and continuous participation of MOHC. However, the adopting behavior was seldom studied, especially from the trust perspective. Thus, the present research tries to fill the gap by exploring the adopting behavior of MOHC from the trust perspective.

### 2.2. Initial Trust

Trust reflects a willingness to be vulnerable that is based on the positive expectation from another party’s future behavior [19]. In general, trust is composed of three beliefs: ability, integrity and benevolence [19]. It has been found that trust has an important effect on users’ adoption in many service contexts, such as m-paying [24], online banking [25], social network [26] and public e-services [27]. With the emerging of mobile medicine in recent years, trust in the field of mobile health is gaining attention. For example, Akte (2013) revealed that perceived trust was positively related to the satisfaction and mHealth services continuance intentions [28]. Guo (2016) examined the mediator role of trust between privacy–personalization paradox factors and mHealth acceptance intention [29]. Mpinganjira (2018) conducted an empirical study to examine the precursor factors of trust in health-related virtual communities context. The result demonstrated that the consumers’ overall trust was significantly influenced by information usefulness, community responsiveness and shared vision [30]. The empirical study from Audrain-Pontevia (2018) found that interpersonal trust in online health community has a positive association with patients’ trust and satisfaction toward their physician [31]. 

According to the formation stage of trust, there are two types of trust including initial trust and cumulative trust [17]. Initial trust is established during the first interaction and occurrs when parties are unfamiliar with each other [32]. With the increase of interaction, when users have gained more experience, initial trust develops into cumulative trust. Previous studies have identified diversified influence factors of initial trust, which can be summarized in four categories. The first category is relevant to personality-based factors, such as trust propensity, which reflect a natural inner tendency of the individual. The prior study indicated that trust propensity is a significant predictor for initial trust [25]. The second category is relevant to performance-based factors, such as the usability of website, information quality and service quality. These factors have been found to have significant effect on initial trust [33]. The third category relates to the online vendors or service providers, such as reputation, company size and willingness to customize. The existing studies also found these factors have a significant positive association with initial trust [13]. The fourth category refers to transfer-based factors. For example, trust in the websites can be transferred from the third parties’ evaluation [34]. For multi-channel context, such as in the web-and-mobile business context, web-based trust can positively affect users’ initial trust in a mobile-based counterpart [35]. Both functional consistency and the trust in web shopping services have positive association with mobile shopping trust [32].

Base on a careful literature review on initial trust, we can find that there are mainly three critical research gaps that provide new opportunities and room for further research. The first gap is that most of the prior studies on online health trust did not distinguish ongoing trust from initial trust [28,29,30,31]. Initial trust, which is developed in a short initial transaction period without prior experience, is significantly different to ongoing trust [36]. Though initial trust is temporary, it can affect whether subsequent interactions occur. Ongoing trust is developed based on the initial trust. The second gap is that the existing studies on online health trust merely focused on overall trust and did not consider more detailed trust elements [28,29,30]. However, independent entities simultaneously participate in the MOHC service providing process. Trust might be attributed to different entities’ effect. Thus, more detailed trust elements should be considered. The third gap is that most of the extant studies separately consider the performance-based [19] and transfer-based [20,21,22] trust building paths. However, the related research shows that both paths have simultaneous effect on initial development, especially for the new emerging online service context [32].

### 2.3. Trust Transfer

Trust transfer refers to formed trust that can be transferred between the trusted entity and the unknown entity based on the association between target entity and trusted source entity [27]. Prior researchers found that trust can be transferred in the following situation. Firstly, when similarity and proximity are perceived between the target and the trusted entity by the trustor, the trust may be transferred. For example, the more functional consistency existing between web and mobile shopping services, the more likely the user will establish the initial trust towards the mobile shopping services. Because the functional consistency reflects the extent of similarity between these two entities [32]. Secondly, when the target and the trusted entity are contextually related or belong together, the trust also may be transferred. For example, the consumers’ trust in one B2C market intermediary may be transferred to the sellers, which may further enhance the consumers’ purchase intention [37]. Thirdly, trust may be transferred when the trustor is influenced by communication and social interaction with others. For example, both interpersonal and public administration recommendations have positive effect on trust in the internet [27]. If the user received positive word of mouth from the trusted or experienced user, the trust may be more easily established. In the present research, trust transfer theory will be used to examine the related relationship between initial trust in doctor and MOHC platform, and their trust transfer-based paths.

### 2.4. Elaboration Likelihood Model

ELM originated from social psychology, which explains how individuals’ attitudes and behaviors change from a dual-path perspective to include central and peripheral paths [38]. The central route refers to the individual carrying out precise cognitive thinking on task-related information and carefully examining the comparative advantage and relevance of this information to form a cognitive judgment on the target behavior. The peripheral route refers to the individual judging target behaviors through simple clues or reasoning, which does not involve deep cognitive thinking [38]. The difference between the central route and the peripheral route is mainly reflected in the following three aspects [38]. Firstly, the information processed by the two routes is different. The central route deals with arguments and clues of the information, while the peripheral route means that individuals deal with some heuristic clues of the information. Secondly, the central route requires careful consideration and understanding of the arguments. In this process, information receivers often invest more cognitive energy, while the information processed by the peripheral route is less demanded, and information receivers only need to consider the obvious clues. Thirdly, the effects of the two paths are different because the central path changes attitudes through hard-working thinking about information-related arguments, so its attitude change is more stable and durable. The peripheral route changes attitudes through information-related heuristic, which leads to the attitude changes are often temporary.

ELM has been widely used in the user behavior study of IS discipline. For example, in mobile shopping services, Yang (2016) applied the ELM to explore initial trust building process [32]. In the travel website context, Tseng and Wang (2016) discovered the effect of perceived risk on individual information adoption behavior based on ELM [39]. Nel and Boshff (2017) employed ELM to examine application-based mobile services trust [40]. Based on ELM, Chen et al. (2018) investigated the influence factors of mobile health applications’ continuance using intention in the developing markets [41]. The prior studies indicated that ELM is an effective theory to explore the basic influence process of users’ cognition and behavior. Thus, we will employ ELM to reveal the development process of initial trust in MOHC context.

## 3. Research Model and Hypotheses

**Figure 1:**
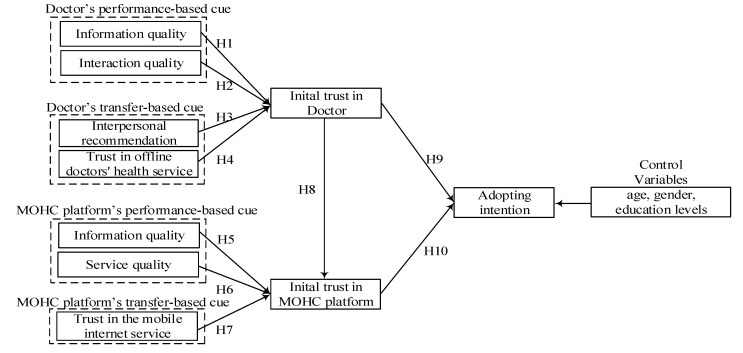
Conceptual model.

According to the trust transfer theory, trust in an entity is composed of different components and is based on the perception of the most significant components [27]. The accumulation or dissolution of trust depends on the effects of cumulative interactions within the various components [27]. The initial trust in MOHC consists of two salient components: initial trust in doctor and initial trust in MOHC platform. Doctors are the main medical service provider. MOHC platform is a medium to implement the online mobile health service. Firstly, we examined the association between initial trust in doctor and MOHC platform, and how these two elements together influence the adopting intention. Secondly, based on ELM and trust transfer theory, a research model was developed to examine the effect of performance-based and transfer-based trust building cues on initial trust in doctor and MOHC platform. Based on the existing studies, we posited that: (1) For the initial trust in doctor, doctor’s information quality and interaction quality as two performance-based cues may positively associate with the initial trust in doctor through central route. Interpersonal recommendation and trust in offline doctors’ health service as two transfer-based cues may positively associate with the initial trust in doctor through peripheral route. (2) For the initial trust in MOHC platform. MOHC platform’s information quality and service quality as two performance-based cues may positively associate with initial trust in MOHC platform through central route. Trust in the mobile internet service as a transfer-based cues may positively associate with initial trust in MOHC platform through peripheral route. At the same time, previous IS researches suggest that individuals’ age, gender, education levels and internet usage experience have been found to affect their using intention toward new system using [42]. Thus, we controlled the effects of these demographic variables on the adopting intention, i.e., age, gender, education levels. The research model is shown in Figure 1.

### 3.1. Doctor’s Initial Trust Formation Process

The doctor’s information includes diseases consultation information between doctors and patients, health science knowledge published by doctors and so on [41]. The quality of doctors’ information reflects the professional competence of doctor, which is helpful to increase the perceived useful of information and the trust between patient and doctor [43]. Unlike ordinary mobile medical systems, many professional doctors are gathered in MOHC to provide disease consultation services for patients. This is also an important feature to attract users. Thus, patients expect to get the relevance, accuracy and adequacy of information from the doctors. If the patient cannot obtain the quality information from the doctor, this will decrease the trust between patient and doctor. Thus, we stated the following:

**Hypothesis** **1.** *Doctor’s information quality is positively related to initial trust in doctor*.

Doctor’s interaction quality reflects the quality of the interaction and dyadic interplay between doctor and patient [44]. Care is one of the core theme of interaction quality which reflects the individualized attention which is provided from the doctor to the patient. In the process of interaction between doctor and patients, doctor not only solves patients’ disease problem but also conveys emotional care to the patient. The emotional care and support reflect the goodwill of doctor, which has significant influence on the patient’s trust toward the doctor [43]. Doctors’ interaction attitude has a significant positive association with the trust between doctor and patient [45]. Meanwhile, a prior study noted affection, including showing concern, encouragement, or care for users, significantly influences their using intention in the mobile-based services context [46]. Thus, we stated the following:

**Hypothesis** **2.** *Doctor’s interaction quality is positively related to initial trust in doctor.*

Risk barrier negatively influence user’s mobile service using intention [47]. Perceived security is vital for the adopting of mobile-based services [48]. The interpersonal recommendation is considered as a more reliable information source than other sources, which is helpful for increasing perceived security and reducing the perceived risk [49]. The positive recommendation of an organization’s service from the colleague, friends or relatives, which reflects the experienced user’s own trustworthiness towards the organization, will diminish the effect of perceived uncertainty [27]. It has been found that interpersonal recommendation is the main factor influence the trust building in prior researches. Such as, Terres and Basso (2018) found that the positive recommendation from the friends, family, healthcare plan and other doctors have a significant effect on initial trust in doctor [50]. Thus, we stated the following:

**Hypothesis** **3.** *Interpersonal Recommendation of the doctor in MOHC from others is positively related to initial trust in doctor.*

Inter-channel trust transfer refers that trust can be transferred in the different contexts, such as from offline to Web channels or from Web to mobile channels. Meng et al. (2019) suggested that trust in offline health services can transfer to the trust in mHealth services [51]. Belanche et al. (2014) noted that the trust in online public can be transferred from the trust in offline public administration recommendation [27]. Lu et al. (2011) reported that the initial trust in mobile-based payment services was affected by the trust in web-based corresponding services [35]. According to the trust transfer theory and prior studies, we posit that the existing trust in offline doctors’ health service experience and familiarity can be transferred to the online initial trust in doctor. Thus, we state the following:

**Hypothesis** **4.** *Trust in offline doctors’ health service is positively related to initial trust in doctor in MOHC.*

### 3.2. MOHC Platform’s Initial Trust Formation Process

In MOHC platform, in addition to health consultation information provided by the doctors, it also includes the information provided by the system platform [7]. For example, health science knowledge, hospital, doctor information, drug information, and self-health examination information and so on. The stronger users’ perception of information accuracy, sufficiency, relevancy and timeliness, the stronger their perception of information quality. The quality of health information on the platform will affect users’ trust and satisfaction toward the platform operation [19]. In addition, accurate health information can popularize health knowledge and help users to carry out reasonable self-health management, while inaccurate information has a negative impact on users’ healthy life. Prior studies found that information quality of system has a significant positive association with the users’ initial trust [19]. Thus, we state the following:

**Hypothesis** **5.** *MOHC platforms’ information quality is positively related to initial trust in MOHC platform.*

Users often expect to obtain efficient and reliable health service from MOHC which is vital for their life and health. The users may form the trust belief towards the platform’s ability to deliver quality services when these expectations are satisfied. However, if users cannot obtain reliable, prompt and personalized health services during the first interaction with the MOHC system, they may feel that the MOHC platform lacks ability to offer qualified health services and cease initial trust. In addition, prior researches have shown that system quality has positive association with the users’ initial trust in various IS context, such as, mobile banking [19], mobile paying [18], mobile shopping [32] and so on, Thus, we state the following:

**Hypothesis** **6.** *MOHC platforms’ service quality is positively related to initial trust in MOHC platform.*

Intra-channel trust transfer refers that the trust can be transferred between the related entities within the same channel. It is advantageous to transfer trust from a known entity to an unknown entity when two entities are related or similar [52]. In the prior studies on the intra-channel trust transfer, Lien (2014) found that patients’ trust in head hospital has a positively effect on their trust in affiliated hospitals [53]. Nel and Boshoff (2017) suggested that the online service trust positively influenced the application-based mobile services trust [40]. Sun et al. (2014) noted that perceived operational consistency and similarity of the linked organizations favors the trust migrates from trusted to unfamiliar entity [52]. MOHC belongs to mobile internet service. It has certain associations, similarities, or operation consistency with other mobile internet services. Based on the trust transfer theory, we stated that the trust accumulated in previous mobile internet services using experience will be transferred to the trust in MOHC platform.

**Hypothesis** **7.** *Trust in the mobile internet service is positively related to initial trust in MOHC platform.*

### 3.3. Relationship Between Doctor’s Initial Trust and MOHC Platform’s Initial Trust

According to the trust transfer theory, interpersonal trust in the members may bring trust in community. The interpersonal trust makes the users feel that they abide by established rules which governed by the community. This enables users to trust the ability of the community to provide effective services and create a trustworthy community environment [54]. Previous studies found that interpersonal trust in the community positively affect the trust in community. Fu et al. (2018) noted that users’ trust toward the members of a community has a positively effect on their trust toward the social community [49]. Zhou (2019) suggested that trust in other members is positively associated with the community trust [5]. Initial trust in doctor represents the interpersonal trust between the the doctors and potential users. MOHC platform represents the professional virtual community for the doctors and patients. According to the trust transfer theory and the existing researches, initial trust in doctor can transfer to the initial trust in MOHC platform. Thus, we state the following:

**Hypothesis** **8.** *Initial trust in doctor is positively related to initial trust in MOHC platform.*

### 3.4. Initial Trust and Adopting Intention

Initial trust is a vital acceptance influence factor. The previous researches have investigated the mechanism of initial trust on reducing uncertainty in technology acceptance and diffusion context [15,17]. Researches suggested that when a new service is not commonly known to the public and involves uncertainty or potential risks, users usually determine whether to adopt this service base on the trust evaluation. In this process, initial trust plays a vital role in eliminate the perceived risk and uncertainty in the interactions [17]. The significant positive association between initial trust and use intention has been verified in prior researches [17,25]. Compared to traditional offline health service, health service conveyed in mobile internet involved more uncertainty and risk. Thus, the initial trust in the MOHC providers which included doctors and platform are critical factors for users to making an adopting decision. Thus, we state the following:

**Hypothesis** **9.** *Initial trust in doctor is positively related to the adopting intention of MOHC.*

**Hypothesis** **10.** *Initial trust in MOHC platform is positively related to the adopting intention of MOHC.*

## 4. Methodology

### 4.1. Measurement

Ten reflective constructs were included in the research model. In order to improve the reliability and validity of measurement, all the items were adapted from the extant literatures which have been pre-validated. We also revised them according to the characteristics of the present research background. To ensure consistency, firstly, one researcher translated all the items into Chinese. After that, another researcher translated them back into English. The final measurement items and references are presented in Table 2. Each construct was measured with three items. Seven-point Likert scale was used to measure each item.

There are three parts in the questionnaire. The first section refers to the introduction of the survey, such as investigation unit, anonymity and confidentiality statement, questionnaire completion method. The second section includes basic demographic information and respondents’ prior using experience of MOHC. This information is important for filtering the respondents. The third section is the measurement items of the construct in the research model.

### 4.2. Sampling Design

University students in eastern China were selected as the respondent. According to Annual Comprehensive Research Report on Internet Medical Treatment in China, young users under 30 years old are the main users of mobile health service, accounting for 36.56% of the total users. It is appropriate to choose students as subject in our research context as they represent the potential young group of mobile health service. These students are undergraduate, graduate student and doctoral students from different majors. In order to evaluate the initial trust building process. The researchers first asked the participants whether they have MOHC using experience. Then, the participant without previous experience were invited to conduct follow-up survey. Before conducting the survey, the researchers introduced the main functions of MOHC. Then, the respondents were required to install the MOHC software to experience the main function. The experience should last no less than 10 min. Finally, the respondents were required to fill out the questionnaire according to their first using experience. We set the function of the questionnaire to avoid missing answers. The questionnaire can only be successfully submitted after all the questions have been answered. All the responses were scrutinized and dropped those with obvious repetition pattern or completed too quickly. The survey was conducted from December 2019 to January 2020. We obtained 301 valid samples which were used for the final analysis. Among them, 65% were female and 35% were male. 73% were ranging from 20–25 years old, 27% were ranging from 26–35 years old. 85% of the respondents are undergraduate, 11% are graduate students, 4% are doctoral students. Most of them (81%) had mobile internet service usage experience more than 3 years. This study has ethics approval from the Academic Committee in School of Management, Hangzhou Dianzi University, Hangzhou, China (Approval number: EC 2019-11-07). Our research also meets the requirement of personal information security protection in the personal privacy protection law (Law no. 2. 1).

## 5. Data Analysis and Result

### 5.1. Measurement Model

Partial least square (PLS) path modeling was used for assessing the research model. Contrast with the covariance-based structural equation modeling (SEM) which require a normal distribution, such as LISREL, the PLS method is a nontraditional component-based SEM approach [55]. It is suitable for the predictive research without requiring normal distribution of the data [56]. Due to the predictive nature of this study and the non-normal distribution character of the data, SmartPLS 2.0 was used to examine the measurement and structural model, which has been widely adopted in the existing researches [49,57,58].

The validity and reliability analyses for each construct were conducted. Convergent validity was tested by item loadings (loadings standard >0.7), average variance extracted (AVE standard >0.5), composite reliability (CR standard >0.7), and Cronbach’s Alpha (CA standard >0.7). As shown in Table 3, all indicators have reached standard values which indicate the convergent validity of all the constructs are acceptable. Discriminant reliability was tested by comparing the square root of AVE and correlation coefficients. As shown in Table 4, each construct’s square root of AVE is greater than its correlations with other constructs, therefore indicating acceptable discriminant reliability.

Following the method which have been used in the past studies [57,59], the multicollinearity was assessed by the value of variance inflation factor (VIF). As shown in Table 3, the highest VIF value of the variable was 2.453, which was below the threshold of 5 [56]. Thus, the multicollinearity was not an issue in the present study.

### 5.2. Common Method Variance Testing

The possibility of common method variance was examined by the following two tests. Firstly, ten factors were extracted by Harman’s single factor test. The largest variance is 17.2%. 

Therefore, most of the variance cannot be explained by any one variable. Secondly, based on the method from Rönkkö and Ylitalo [60], three items of internet technicality which had low correlations with other items in the present research model is used as marker variable items. Internet technicality were collected in the same survey. Then, a method factor model was created as follows: the marker variable as the exogenous variable was included in the model to predict each endogenous latent construct. Finally, comparing the baseline model and method factor model, we found that the hypothesized relationships in the baseline model was still significant in the method factor method. These test results indicate that common method bias will not significantly influence this quantitative research.

### 5.3. Structural Model

**Figure 2:**
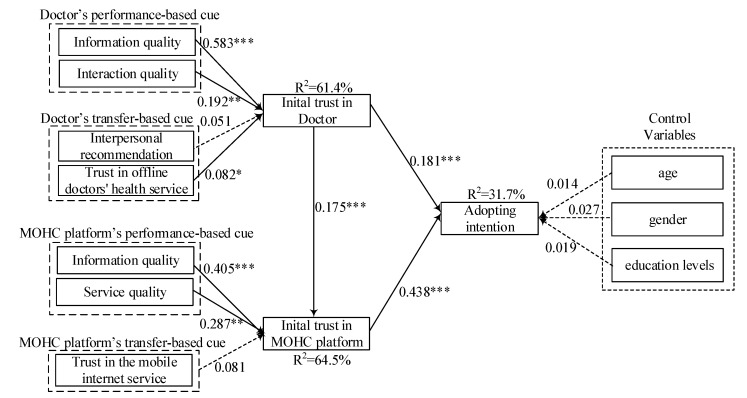
**Structural Model.** (Note: * *p* < 0.05; ** *p* < 0.01; *** *p* < 0.001).

R^2^ of the dependent and the path coefficients of the hypotheses were measured as shown in Figure 2. As expected, both of doctor’s information quality and interaction quality are positively related to the initial trust in doctor, in support of H1, H2. Trust in offline doctor’s health service is positively related to the initial trust in doctor, in support of H3. MOHC’s information quality and service quality are positively related to initial trust in MOHC platform, in support of H5, H6. Initial trust in doctor is positively related to initial trust in MOHC platform, in support of H8. Regarding the predictors of adopting intention, both initial trust in doctor and initial trust in MOHC platform are positively related to adoption intention, in support of H9, H10. On the other hand, interpersonal recommendation and trust in the mobile internet service have insignificant association with initial trust in doctor and MOHC platform. Therefore, H3, H7 are not supported. In additional, three control variables (age, gender and education levels) have insignificant influence the adoption intention. This result partly consists with the study of Duarte and Pinho (2019) [10]. Both present study and Duarte and Pinho (2019) [10] found the age and gender insignificantly influence the adopting intention in mHealth context. Different with the present study, the study of Duarte and Pinho (2019) [10] found education has significant effect on adoption intention. The possible reason may be the nearly 72% of the sample in Duarte and Pinbo (2019) [10] are graduate or post-graduate students. However, 85% of the sample in our study are undergraduates. This different research result also shows that when the overall educational level of the sample increases, the effect of educational level on the adoption intention will show up. Conversely, when the educational level of most samples is concentrated in a relatively low level, the impact of education level on adoption intention is insignificant.

Doctors’ information quality and interaction quality explain 61.4% of the variance of initial trust in doctor. MOHC platform’s information and service quality explain 64.5% variance of initial trust in MOHC platform. Initial trust in doctor and initial trust in MOHC platform explain 31.7% variance of adopting intention.

## 6. Discussion

### 6.1. Key Finding

The result offers several key findings. Firstly, we propose that initial trust in MOHC comprises two significant interrelated elements that influence the adopting intention. The data analysis result is consistent with the hypotheses and demonstrates that both initial trust in doctor and mobile MOHC platform are positively related to adopting intention. It is noticeable that initial trust in MOHC platform is more critical for adoption intention (β = 0.438, *p* < 0.001). Even though the initial trust in doctor also has positive association with the adoption intention, its effect is limited (β = 0.181, *p* < 0.001). This may be for the reason that for first-time users of MOHC, the medical services they previously received are mainly offline. MOHC has changed medical service delivery channels; thus, the users will pay more attention to this new underlying technology provider. In other words, for the unexperienced users of MOHC, the risk perception mainly comes from the platform rather than the doctor. Therefore, the initial trust in MOHC will exert greater association with adopting intention. In addition, initial trust in doctor has significant and positive association with the initial trust in MOHC platform (β = 0.175, *p* < 0.001). This result aligns with the trust transfer theory [22], which implies that the interpersonal trust between the users and doctor in MOHC can transfer to the MOHC platform. The doctor is the core health service provider in MOHC, which represents the professional medical level of the platform to a certain extent. Thus, the initial trust in doctor is helpful for the formation of initial trust in MOHC.

Secondly, the present research respectively examines the roles of performance-based and transfer-based trust building cues in forming the initial trust in doctor and MOHC platform. For the initial trust in doctor, the results indicated that performance cue (e.g., information quality (β = 0. 583, *p* < 0.001) and interaction quality (β = 0.192, *p* < 0.01)) and transfer-based cue (e.g., Trust in offline doctors’ health service (β = 0.082, *p* < 0.05)) have a significant and positive association with initial trust in doctor. Information quality and interaction quality have relatively larger effects. To some extent, information quality of doctor reflects the ability, that represents the professional competence of a doctor. Meanwhile, the interaction quality reflects the benevolence, which represents the doctor’s concern for the patient. Both ability and benevolence are the core components of trust. Thus, information quality and interaction quality of doctor are the key factors related to the initial trust in doctor. At the same time, the result is consistent with a prior study [51]; trust in offline doctors’ health service has significantly positive association with initial trust in doctor which implied that trust accumulated over time in the offline health context indeed has a cross-environment effect on the initial trust in mobile-health context.

For MOHC platform, only performance-cues that included information quality (β = 0.405, *p* < 0.001) and system quality (β = 0.278, *p* < 0.01) have significant positive association with initial trust in MOHC, and both of these two factors explain the 64.5% variance for initial trust in MOHC. Initial trust in MOHC platform cannot be formed from trust-transfer based cue. This result suggested that the user will invest more effort and resources to access information quality and service quality for establishing the initial trust in MOHC. On the one hand, in line with the prior study, information quality has been found to be a significant factor positively related to the initial trust in mobile APP platform [19]. MOHC provides an abundance of information about health knowledge, disease self-diagnosis information, online physician, hospital information and so on. The users expect to obtain accurate, timely and relevant information in the MOHC. For example, the registration information of the doctors in MOHC should be synchronous with the offline hospital. Otherwise, users may obtain the wrong registration information when they book the health service via MOHC, which may damage the trust in MOHC. On the other hand, service quality also exerts significant impact on initial trust in MOHC. This suggested that the users expect to obtain personalized and reliable services. For example, the platform can offer personalized health service according to the users’ browsing history and ensure that users can receive prompt medical advice after payment.

Thirdly, in contrast with our assumptions, H3 and H7 are not supported. The potential reasons are the following: (1) The online diagnosis from the doctors is mainly based on the communication between doctors and patients or the existing medical reports, they cannot make detailed face to face medical observation and judgment, which is an important difference between online medical treatment and offline medical treatment. Therefore, for the new MOHC users, they may be worried about doctors’ diagnosis because of these limitations. These doubts reduce the power of interpersonal recommendation. (2) Unlike other mobile internet services, such as mobile banking, e-public service and so on, the risk perception is much stronger in the case of medical health services, which is related to users’ health and safety. Thus, the existing trust in the mobile internet service cannot transfer to the initial trust in MOHC platform. The users prefer to use the performance cue that they truly experienced rather than the transfer-based cue.

### 6.2. Theory Implications

From the theory perspective, the present study contributes to the existing literature as follows.

Firstly, this study contributes to the MOHC user behavior research and mHealth adopting studies by highlighting the effect of initial trust on users’ adoption. As noted earlier, the extant researches have explored the effect of technological factors, individual characteristics, value factors, environmental factors and psychological factors on mHealth user adoption behavior based on the theory of UTAUT, TAM, VAB and TPB [2,4,9,10,11,12,13,14]. Seldom, the studies explored the role of initial trust on user behavior. However, the initial trust is crucial for the new user adoption, especially for the emerging IS context. Thus, our work extended the MOHC user behavior researches and mHealth adopting studies by proposing an initial trust model for the MOHC adoption.

Secondly, the present study enriches the trust studies in mobile health context by analyzing different trust elements. Most of the prior studies examined the overall trust in the mobile health [28,29,30], few studies have investigated the different trust subjects which will prevent us from forming a comprehensive understanding of the trust building process. Our work shows that initial trust in both provider characteristics (doctors) and technology characteristics (MOHC platform) are the two main elements for the forming of initial trust in MOHC. The effect of initial trust in the MOHC platform on adopting intention is greater than that of the doctor, which implies that the technological delivery channel is relatively more important when the customer decides whether to use the MOHC in the early stage of use. We provide an understanding of how various trust antecedents may operate in online health community initial trust-building process. These findings will enrich the online health trust literatures by analyzing the initial trust [30].

Thirdly, the present study advances trust formation study by simultaneously examining the effects of the transfer-based and performance-based cues. Previous studies separately examined the performance-based trust building process [19] and the transfer-based trust building process [20,21,22]. These existing studies have not examined the simultaneous effects of the transfer-based and performance-based trust building mechanisms on consumer initial trust development, especially in an emerging MOHC context. This study bridged these two important trust building processes based on ELM which facilitate the theoretical understanding of the initial trust antecedents.

### 6.3. Practical Contributions

The present study was conducted in China, according to the Annual Comprehensive Report on China’s Internet Healthcare, low public trust is the main reason why users do not adopt mobile healthcare [61]. Thus, the conclusions of this study and the following practical suggestions can provide reference to the other countries with similar problems in the development of MOHC. From the practical perspective, the findings suggest that MOHC managers should establish users’ initial trust towards both doctor and platform for the promoting of MOHC adoption and usage. The details are as follows:

Firstly, for the MOHC managers, the information quality and interaction quality of the doctors should be ensured. When the users initially interact with the doctors in the MOHC, they will perceive the quality in terms of the competence of the doctors, promptness in providing disease diagnosis and individual care for users’ needs. Thus, on the one hand, the doctors should be screened to ensure their professional competence. On the other hand, the doctor should create a friendly and supportive atmosphere in the process of online consultation. Doctors’ attitude towards patients should be an important indicator of doctors’ evaluation in the system.

Secondly, the trust in offline doctor’s health service can be used as an enabler in building the initial trust in doctors in the MOHC. MOHC managers can invest more resources in attracting the users who have already accumulated a certain level trust in offline doctor. For example, MOHC managers can pay more attention to the patients with chronic diseases who need frequent follow-up visiting, especially those who already have a long-term follow-up relationship with a specific doctor. MOHC managers can encourage them to use their doctor’s online services. Because members of this user group have already accumulated a certain level of trust with their doctors in offline interactions in the early stage, it is easier for them to transfer trust to the online platform and adopt their doctors’ online medical services. At the same time, MOHC managers can also increase the cooperation with doctors that are widely trusted by patients. For example, MOHC can organize for some famous doctors to carry out online free consultation activities. When users find the doctors they have known or trusted in the MOHC, they are more likely to transfer their offline accumulated trust to online.

Thirdly, based on the result, we found that the initial trust in the MOHC was mainly formed by performance-based cue rather than transfer-based cue. Thus, the MOHC managers need to adopt effective strategies to encourage the new users to use it and help them to establish the initial trust based on their real experience. There is a pressing need to improve the information quality and service quality of the MOHC platform. One the other hand, to improve the information quality, MOHC managers should present relevant, useful and accurate information to users. In contrast to the information from the doctor, the information provided by the platform should cover a wider scope, not only including the information about cooperating doctors, hospitals and user personal service information but also including the health science knowledge, patient feedback information, drug information, and so on. The improvement of information in the platform required continuous resources and effort investment from the service provider. The case-based reasoning system [59,62,63] and user profiling technology [64,65] can be integrated into the MOHC platform, which is helpful in providing targeted health information according to users’ needs. On the other hand, the managers of MOHC platform can improve the users’ perception of service quality through personalized service, privacy protection, service guarantee and so on. That is conducive to reduce the perceived risk and build the initial trust. At the same time, as a professional medical diagnosis and treatment platform, undertaking public medical services also has a certain effect on establishing the initial trust and promoting the adoption of users. For example, at the end of 2019 in China, during the outbreak of novel coronavirus pneumonia, some MOHC platforms attracted a great number of new users through free online consultations and the release of professional information. By doing this, it not only helps to alleviate the epidemic but also benefits from shaping a good public image and further establishing users’ initial trust. 

## 7. Conclusions

As an emerging online health service, MOHC has not been widely adopted by users. Due to the higher perceived uncertainties and risk perceptions of mobile health service, building users’ initial trust is important for promoting their adoption intention. Based on the ELM and trust transfer theory, the present study explored the simultaneous effect of performance-based and transfer-based cues on the initial trust building process in the MOHC context and addressed two research questions.

RQ1 examined the effect of two different MOHC initial trust elements on the user’s adopting intention and how they are interrelated. The result indicated that both initial trust in doctor and platform are related to the adopting intention. Especially, the initial trust in MOHC platform exerts a greater effect. Meanwhile, the initial trust in doctor has significant positive effect on initial trust in platform.

RQ2 respectively investigated the two MOHC initial trust elements forming paths based on the performance-based and transfer-based cues. The finding found that the performance-based cues including the doctors’ information quality and service quality in combination with transfer-based cues including trust in offline doctors’ health service are significantly positively associated with initial trust in doctor. However, the transfer-based cues including interpersonal recommendation are insignificantly related to initial trust in doctor. In addition, only performance-based cues including MOHC platform’s information quality and service quality have significant positive association with initial trust in MOHC platform. The transfer-based cue including trust in mobile internet service has insignificant association with initial trust in MOHC platform.

### 7.1. Study Limitations

There are still some limitations in the present study. Firstly, students were chosen as respondents in the present study. Although the student group represents the main user group of the potential MOHC users, they cannot represent all age groups. Especially, in an aging society, older age groups who have higher health risk are also important potential users. Therefore, whether the findings are still robust in other age groups needs further verification. Secondly, the present study mainly focused on capturing the most significant influence factors in the forming process of initial trust. Some other antecedent factors that may also influence initial trust were not all embraced in the model, such as the individual’s personality characteristics including health literacy, trust propensity and so on. Other factors’ possible impacts need to be further validated through an integrated model. Thirdly, the present study was conducted in China and the MOHC is still in its infancy phase; the generalizability of the findings in the present study should be further verified in other countries. Fourthly, this study only examined the adopting intention. However, adopting is the first step for success; the continuance of using behavior determining the long development should be further explored.

### 7.2. Future Work

There are some recommendations to address these limitations in further studies, accordingly. Firstly, the other age groups, such as middle-aged users and senior users, can be included as respondents in future research. The present research model can be replicated to compare the initial trust formation among different age groups when they adopt MOHC. Secondly, in future research, the other influence factors, such as the health literacy, trust propensity and so on, can be integrated into the model as the moderating variable or independent variable to verify the effect of these factors on initial trust and adopting intention. Thirdly, further study can be conducted in other countries which have different cultural characteristics. In this way, we can compare the influence of cultural factors on the formation of users’ initial trust in MOHC context and verify the generalizability of the findings. Fourthly, future study can extend the present study to examine the continuance of using behavior. The existing study suggest that some value factors, such as functional value and social value, are the significant influence factors in user’s continuance using behavior [66]. Thus, the future research can draw on the existing research to examine the impact of these factors on the user’s continuance using behavior in the context of MOHC.

## Figures and Tables

**Table 1:** Summary of the mobile health adopting research.

Source	Theoretical Framework	Significant Key Factors Affecting the Adoption
Alam et al. (2020) [2]	UTAUT + perceived reliability, price value	performance expectancy, effort expectancy, social influence, facilitating conditions, perceived reliability, price value
Duarte and Pinbo (2019) [10]	UTAUT + hedonic motivations, price value, habit	performance expectancy, effort expectancy, social influence, facilitating conditions, hedonic motivations, price value, habit
Quaosar et al. (2018) [13]	UTAUT + perceived credibility	performance expectancy, effort expectancy, social influence, perceived credibility
Hoque and Sorwar (2017) [11]	UTAUT + technology anxiety, resistance to change	performance expectancy, effort expectancy, social influence, technology anxiety, resistance to change
Lee et al. (2017) [14]	Content value, Context value	content value: usefulness, convenience; context value: health stress, epistemic value
Hoque and Raki bul (2016) [4]	TAM + subjective norm, personal innovativeness in IT	perceived ease of use, perceived usefulness, subjective norm, gender
Hsiao andTang (2015) [9]	TAM + perceived ubiquity, health knowledge, health care need, subjective norm	perceived ease of use, perceived ubiquity, health knowledge, health care need, subjective norm
Deng et al. (2014) [12]	value attitude behavior model (VAB), theory of planned behavior (TPB)	For the middle-aged group: perceived value, attitude, perceived behavior control and resistance to change; for the older group: perceived value, attitude, perceived behavior control, technology anxiety and self-actualization need.

**Table 2:** Variable measurement and source.

Construct	Operational Definition and Measurement Items	Sources
Doctor’s information quality (DIT)	The usefulness, sufficiency and accuracy of the information offered by the doctor in MOHC	
	DIT1: The doctor provides me with useful health information	[41,44]
	DIT2: The doctor provides me with sufficient health information	
	DIT3: The doctor provides me with reliable health information	
Doctor’s interaction quality (DIQ)	The caring and individualized attention from the doctors to the MOHC users	
	DIQ1: The doctor understands my specific needs	[41,44]
	DIQ2: The doctor has my best interests at heart	
	DIQ3: The doctor gives me individual care	
Interpersonal recommendation (IR)	The informal or non-commercial information exchanges about the doctors in the MOHC between the potential user and other professionals, friends or relatives	
	IR1: My family recommend the use of MOHC to me	[27]
	IR2: My colleagues recommend the use of MOHC to me	
	IR3: My friends recommend the use of mobile MOHC to me	
Trust in offline doctors’ health service (TODHS)	The users’ belief in the integrity and benevolence of the offline doctors’ health service	
	TODHS1: I trust the doctor who I am consult can provide effective treatment in the offline hospital	[50,51]
	TODHS2: I think I can trust the doctor I’m about to consult in the offline hospital	
	TODHS3: The doctor I am about to consult should probably be very reliable in the offline hospital	
MOHC platform’s information quality (MIQ)	The timeliness, sufficient and relevance of the information offered by the MOHC platform	
	MIQ1: This MOHC platform provides me with information relevant to my needs	[19]
	MIQ2: This MOHC platform provides me with sufficient information	
	MIQ3: This MOHC platform provides me with up-to-date information	
MOHC platform’s service quality (MSQ)	The reliability, efficiency and personalized of the service offered by the MOHC platform	
	MSQ1: This MOHC platform provides prompt services	[19]
	MSQ2: This MOHC platform provides personalized services	
	MSQ3: This MOHC platform provides dependable services	
Trust in the mobile internet service (TMIS)	The users’ belief in the integrity and benevolence of the mobile internet service	
	TMIS1: I trust the mobile internet service	[27]
	TMIS2: The mobile internet is a reliable mean to carry out transactions	
	TMIS3: When making transactions the mobile internet is trustworthy	
Initial trust in Doctor (ITD)	The users’ initial belief in the competence, integrity and benevolence of the doctor in the MOHC	
	ITD1: I believe the doctors in the MOHC platform have medical qualifications	[3]
	ITD 2: The consultation or diagnosis provided by the doctors in MOHC platform is reliable	
	ITD 3: In my opinion, the doctors in the MOHC platform are trustworthy	
Initial trust in MOHC platform (ITM)	The users’ initial belief in the ability, integrity and benevolence of the MOHC platform	
	ITM1: This MOHC platform can fulfill its tasks	[19]
	ITM2: This MOHC platform will keep its promises	
	ITM3: This MOHC platform will keep the customers’ best interests in mind	
Adopting intention (AI)	The users’ using intention of MOHC in the further	
	AI1: I intend to use MOHC to consult health issues when needed in the future	[3]
	AI2: I predict that I will use MOHC to consult health issues when needed in the future	
	AI3: I plan to use MOHC to consult health issues when needed in the future	

**Table 3:** Item loadings, AVE, CR, and VIF values.

Construct	Indicator	Factor Loading	AVE	Composite Reliability	VIF
Doctors’ information quality	DIT1	0.877	0.798	0.921	1.423
	DIT2	0.906			
	DIT3	0.895			
Doctors’ interaction quality	DIQ1	0.843	0.747	0.900	1.531
	DIQ2	0.876			
	DIQ3	0.873			
Interpersonal recommendation	IR1	0.915	0.877	0.956	1.613
	IR2	0.951			
	IR3	0.943			
Trust in offline doctors’ health service	TODHS1	0.891	0.782	0.915	1.286
	TODHS2	0.876			
	TODHS3	0.886			
MOHC platform’s information quality	MIQ1	0.845	0.730	0.890	2.302
	MIQ2	0.854			
	MIQ3	0.862			
MOHC platform’s service quality	MSQ1	0.857	0.721	0.886	1.739
	MSQ2	0.828			
	MSQ3	0.861			
Trust in the mobile internet service	TMIS1	0.869	0.770	0.910	1.468
	TMIS2	0.890			
	TMIS3	0.872			
Initial trust in Doctor	ITD1	0.866	0.819	0.931	1.936
	ITD2	0.936			
	ITD3	0.911			
Initial trust in MOHC platform	ITM1	0.844	0.724	0.887	2.453
	ITM2	0.898			
	ITM3	0.809			
Adopting intention	AII	0.911	0.803	0.924	1.205
	AI2	0.915			
	AI3	0.861			

**Table 4:** Latent variable correlation matrix: Discriminant validity.

Construct	1	2	3	4	5	6	7	8	9	10
1. DIT	0.893									
2. DIQ	0.36	0.864								
3. IR	0.39	0.46	0.936							
4. TODHS	0.18	0.43	0.39	0.884						
5. MIQ	0.42	0.35	0.35	0.36	0.854					
6. MSQ	0.23	0.37	0.38	0.37	0.42	0.849				
7. TMIS	0.34	0.51	0.28	0.34	0.54	0.43	0.877			
8. ITD	0.45	0.48	0.43	0.36	0.51	0.23	0.31	0.905		
9. ITM	0.27	0.41	0.43	0.31	0.39	0.38	0.25	0.30	0.851	
10. AI	0.37	0.32	0.31	0.02	0.32	0.30	0.30	0.38	0.30	0.896
